# Draft Genome Assemblies of Two Cryptosporidium hominis Isolates from New Zealand

**DOI:** 10.1128/MRA.00363-21

**Published:** 2021-07-01

**Authors:** M. A. Knox, J. C. Garcia-R, D. T. S. Hayman

**Affiliations:** a Molecular Epidemiology and Public Health Laboratory, Hopkirk Research Institute, Massey University, Palmerston North, New Zealand; University of Maryland School of Medicine

## Abstract

Cryptosporidium hominis is a protozoan parasite that causes gastrointestinal disease in humans worldwide. Here, we report on draft whole-genome sequences of two clinical isolates of C. hominis that were purified from patients with cryptosporidiosis in New Zealand.

## ANNOUNCEMENT

The protozoan parasite *Cryptosporidium* is an important cause of diarrheal disease in humans and animals, with life-threatening effects in children and immunocompromised people. Cryptosporidium hominis and Cryptosporidium parvum are the most common species causing disease in humans worldwide. C. hominis is predominantly a human pathogen, and thus, transmission is mainly anthroponotic, while that of C. parvum is mainly zoonotic (1). The objective of this work was to sequence human isolates of C. hominis from New Zealand, providing the opportunity to compare New Zealand isolates with those found elsewhere.

The most common C. hominis subtype in human cases in New Zealand is IbA10G2 (2). We sequenced two human isolates of C. hominis at the Hopkirk Institute, Massey University (Palmerston North, New Zealand). This subtype is found globally and has been related to both outbreaks and sporadic infections in industrialized nations (3, 4). Oocysts were semipurified from stool samples using a Ficoll gradient method (5) and were further purified by immunomagnetic separation using a Dynabeads GC-Combo kit (Thermo Fisher Scientific, Vilnius, Lithuania). DNA was extracted with the Quick-DNA fecal/soil microbe miniprep kit (Zymo Research, Irvine, CA, USA), and libraries were prepared using a Nextera XT library preparation kit (Illumina, San Diego, CA, USA) before Illumina HiSeq 2 × 150-bp sequencing. Totals of 719.5 and 675.8 Mb, representing 2.4 million and 2.3 million reads, respectively, were obtained from the sequencing for isolate 1 and isolate 2, respectively. The quality of the reads was examined using FastQC (6). Reads were mapped against a reference C. hominis isolate (GenBank accession number CXWB00000000.1) using Bowtie 2 v.2.4.1 (7), and consensus sequences were generated using BCFtools (8). Reads were then *de novo* assembled using SPAdes v.3.15.0 (9) with the --trusted-contigs flag directed to Bowtie 2 consensus sequences. Descriptions of the resulting draft genome assemblies are summarized in Table 1. To further assess genome quality, we undertook annotation of each genome with the online tool Companion (10) (http://companion.gla.ac.uk) using C. hominis TU502 (GenBank accession number GCA_000006425.2) (11) as a reference. Resulting genome statistics are shown in Table 1.

**TABLE 1:** Genome statistics from SPAdes and subsequent companion analyses

Parameter	Value for:	
	Isolate 1	Isolate 2
GenBank accession no.	JAGGCV010000000	JAGGCU010000000
BioSample accession no.	SAMN18394528	SAMN18394529
SRA accession no.	SRX10463636	SRX10463637
BioProject accession no.	PRJNA716090	PRJNA716090
No. of contigs	38	43
Total genome size (Mb)	9.070	9.061
Coverage (×)	58.6	53.6
*N*_50_ (kb)	635.9	539.6
Size of largest contig (Mb)	1.028	0.878
No. of genes	3,840	3,838
Gene density (genes/Mb)	416.93	417.18
No. of coding genes	3,781	3,780
No. of pseudogenes	44	43
No. of genes with function	2,331	2,331
No. of pseudogenes with function	23	22
No. of noncoding genes	59	58
No. of genes with multiple coding sequences	4	2
Overall GC content (%)	30.12	30.13
Coding GC content (%)	31.75	31.76

### Data availability.

Accession numbers are available in Table 1.
